# Donor heart selection: the outcome of "unacceptable" donors

**DOI:** 10.1186/1749-8090-2-13

**Published:** 2007-02-17

**Authors:** Noman H Khasati, Ali Machaal, Jim Barnard, Nizar Yonan

**Affiliations:** 1 Cardiothoracic Surgery Department, Wythenshawe Hospital, Manchester, UK

## Abstract

**Background:**

The decline in the number of suitable donor hearts has led to an increasing interest in the use of previously unacceptable donors. In the United Kingdom, if one centre declines a donor heart on medical grounds it may be offered to other centres. This multi-centre study aimed to evaluate the outcome of recipients of donor hearts considered medically unsuitable for transplantation by one centre that were used in other centres.

**Methods:**

Between April 1998 and March 2003, ninety-three donor hearts (group A) were transplanted, after being considered medically unsuitable for transplantation by another centre. During the same period, 723 hearts (group B) were transplanted in the UK using donors not previously rejected. Data on the donors and recipients was obtained from the UK transplant database. Comparative analysis on the two groups was performed using SPSS 11.5 for Windows.

**Results:**

The characteristics of recipients were similar in both groups. The main reasons for refusal of hearts are listed below. In most cases there was more than one reason for refusing the donor heart. We did not find significant differences in the post-operative mortality (up to 30 days), ICU and hospital stay and cardiac cause of death between the two groups. Kaplan-Meier survival curves showed no significant difference in the long-term survival, with Log Rank test = 0.30.

**Conclusion:**

This study demonstrates that some hearts declined on medical grounds by one centre can safely be transplanted and should be offered out nationally. The use of these hearts was useful to expand the scarce donor pool and there does not seem to be a justification for denying recipients this extra source of organs.

## Background

Using donor hearts outside the conventional selection criteria provides a useful expansion to the donor pool. The data on the use of borderline hearts was not unequivocal. Several studies [1-5] have shown that survival with marginal hearts can be similar to that obtained with standard donor hearts. Other studies [6-10] however, showed increased early or late postoperative mortality and morbidity. A study of recipients of donor hearts declined by our centre on medical grounds and used by another centre showed that the survival was lower than standard donors [6]. This retrospective multi-centre study aimed to evaluate the outcome of recipients of donor hearts considered medically unsuitable for transplantation by one centre, but then were used by other centres in the United Kingdom.

## Patients and methods

The selection of the donor heart can sometimes be difficult and subjective despite the guidelines for acceptance of cardiothoracic organs (Table 1). If one centre considers a donor heart medically unsuitable, it may be offered to other centres through UK Transplant using the relevant Cardiac or Lung Centre Rota [11], but it is generally viewed with caution. In this study, adult heart recipients undergoing heart transplantation between April 1998 to March 2003 were analyzed. We separated recipients into 2 groups based on the donor profiles. Group A consisted of recipients of donor hearts considered medically unsuitable for transplantation by one centre, but then accepted and transplanted in another centre in the UK. Group B comprised patients, who received donor hearts not previously rejected. Donor and recipient information was extracted from the United Kingdom Cardiothoracic Transplant Audit database. The post-operative mortality (up to 30 days), ICU stay, hospital stay and survival, were studied in both groups. Donor and recipient characteristics were compared with the X^2 ^test (categorical data) or the Kruskal-Wallis test (continuous variables). All mean values are expressed ± the standard deviation from the mean. Survival curves were estimated using standard Kaplan-Meier actuarial analysis. We defined significance as p ≤ 0.05. We performed statistical analysis using SPSS 11.5 for Windows.

**Table 1:** Donor heart suitability criteria

Age	Up to 65 years
Size compatibility	Height ± 15%, weight ± 25%
Inotropic support:	Dopamine 10 mcgs/kg/min or Noradrenalin 10 ml/hr (4 mg in 50 mls)
Filling pressures	Normal BP, CVP10, PAWP 15
ECG	No LVH, No left axis deviation, ST/T changes can appear following BSD
CXR	Normal cardiac size, contour and normal cardiothoracic ratio
Infections	Acceptable if treated with the appropriate antibiotics. Viral meningitis is not acceptable. HIV-contraindication, Hepatitis C – acceptable for Hep C positive recipients
Past Medical History	Angina, Hypertension, Hypercholesterolemia-contraindications
Smoking	Acceptable up to 20 pack years
Cardio toxic drugs	No intake of amphetamines, cocaine, and tricyclic antidepressants
Tumours	Brain tumours may be acceptable according to type

## Results

In the period from April 1998 to March 2003, ninety-three donor hearts (group A) were transplanted in the UK, after being considered medically unsuitable for transplantation by another centre. During the same period, 723 hearts (group B) were transplanted in the UK using donors not previously rejected.

The characteristics of recipients were similar in both groups (Table 2). Recipient age was 49.7 ± 10.5 and 47.14 ± 11.9 for groups A and B respectively. Ischemic heart disease was present in 40% of the patients in group A and 35 % of patients in group B. The severity of the recipient condition (NYHA class, being in hospital, the use of Inotropes and intraaortic balloon pump) was similar in the two groups. Only 1% of the recipients in each group had Left Ventricular Assist Device preoperatively (one patient in group A and 8 in group B). The percentage of patients with Donor/Recipient weight ≤ 0.7 was 15% in group A and 38% in group B. Donor hearts refused primarily because of the size mismatch were not included in group A. The ischemic time was 217.09 ± 53.1 for group A and 191.43 ± 57.7 for group B.

**Table 2:** Recipient Characteristics.

	**Group A**	**Group B**
Recipient age (y)	49.7 ± 10.5	47.14 ± 11.9
Recipient BMI	25.2 ± 4.8	25.7 ± 4.2
Donor/Recipient weight ≤ 0.7	15% of the cases	38 % of the cases
Ischemic time (min)	217.09 ± 53.1	191.43 ± 57.7
IHD recipient (%)	40	35
Recipient in hospital (%)	19	22
Recipient IABP (%)	3.3	4.4
Recipient in NYHA 4	31.4%	30.8%

BMI – Body Mass Index; IHD – Ischemic Heart Disease; IABP-Intra-Aortic Balloon Pump.

**Figure 1:**
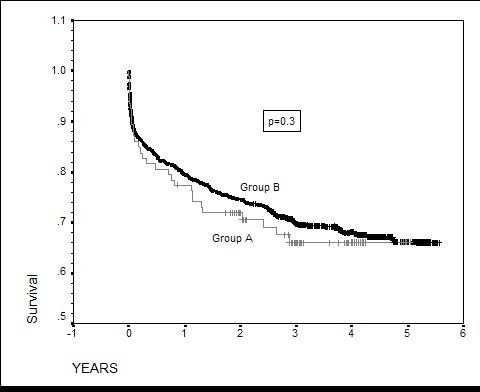
Kaplan – Meier curves. Group A – recipients of " unacceptable hearts", Group B – recipients of standard donors.

The main reasons for refusal of hearts are listed in Table 3. In most cases there was more than one reason for refusing the donor heart. Coronary Angiography and Echocardiography was not performed routinely, and we do not have any data regarding a refusal based on echocardiographic information. There was no significant difference in the 30-day mortality (13.6 % in group A versus 12.9 in group B); ICU and hospital stay (2.3 ± 2.9 and 25.3 ± 15.7 in group A and 4.1 ± 8.5 and 26.0 ± 17.6 in group B respectively) between the two groups. 30% of the patients in group A and 22% in group B recipients died of cardiac causes (Table 4). Early graft dysfunction was the primary cause of death in 75% of cardiac deaths in group A and 69% in group B. Kaplan-Meier survival curves showed no significant difference in the long term survival, with Log Rank test = 0.30 (fig. 1)

**Table 3:** Reasons Why Hearts Were Declined by first centre (n = 93).

	Primary Reason	Secondary Reason
Inotropic Support * (%)	23.6	4.3
Haemodynamic Instability^†^(%)	10.7	8.6
ECG Changes^‡ ^(%)	10.7	5.3
Age^§ ^(%)	5.3	12.9
Past Medical History (%)	16.1	13.9
Abnormal CXR^+ ^(%)	4.3	3.2
Smoking ^□ ^(%)	6.5	38.7
Other^# ^(%)	22.8	13.1

* Dopamine >10 μg/kg/min or noradrenalin >0.2 μg/kg/min or adrenaline >0.5 μg/kg/min.

^† ^High filling pressures and low systemic blood pressure.

^‡ ^Abnormal rhythm, bundle branch block, or ST wave changes.

^§ ^Up to a maximum of 65 years.

^+ ^Abnormal cardiac size/cardiothoracic ratio or pulmonary oedema.

^□ ^Up to 20 pack-years (i.e. 1 pack/d for 20 years).

^# ^Cerebral astrocytoma grade IV, brain tumour with unknown histological findings, hypernatremia and hyperkalemia of unknown cause and significant history of drug abuse.

**Table 4:** Outcome.

	Group A	Group B	P
30 day mortality	13.6	12.9	0.5
ICU stay	2.3 ± 2.9	4.1 ± 8.5	0.07
Hospital stay	25.3 ± 15.7	26.0 ± 17.6	0.2
Cardiac cause of death	30%	22%	0.4

## Discussion

All cardiothoracic organs donated in the United Kingdom or Republic of Ireland is offered to the transplant units according UK Transplant Organ Sharing Scheme Operating Principles for Cardiothoracic Transplant Units in the UK and Republic of Ireland. The selection of the donor hearts can be difficult and subjective. The decline in the number of suitable donor hearts has led to an increasing interest in the use of previously unacceptable donors. The data on the use of borderline hearts was not unequivocal. Several studies [1-5] have shown the safety of the use of borderline donor hearts, especially if invasively evaluated and resuscitated. Other studies [6-10] however showed increased early or late postoperative mortality and morbidity. A multi centre study by the United Kingdom Cardiothoracic Transplant Audit steering group showed a definite effect of suboptimal donor quality with 12% decrease in 1-year survival[7]. A study by Hetzer group from Berlin, found a significant increase in long-term cardiac morbidity due to more focal coronary stenosis in group of recipients of older donors8. Topkara et al found that increased donor age is an independent predictor of reduced long-term survival[9]. A retrospective study of recipients of donor hearts declined by our centre showed that the survival was lower than standard donors[6].

The shortage of organs necessitates pushing the boundaries of the guidelines. This has translated into harvesting of older donor hearts, from more unstable donors as well as from more distant locations. Of utmost importance is that when the decision is made to proceed with cardiac transplantation, the risk/benefit ratio associated with cardiac transplantation in that particular patient must be weighed against the mortality and morbidity risk while remaining on the heart transplant waiting list. Therefore, decisions need to be made on an individual basis. Despite the liberation of the criteria for suitability for donation, many surgeons continue to be conservative in accepting potentially usable organs. If one centre considers a donor heart medically unsuitable, it may be offered to other centres through UKT using the relevant Cardiac or Lung Centre Rota, but it is generally viewed with caution. Some surgeons transplant borderline hearts to high-risk recipients, because they feel high-risk recipients have the greater marginal benefit from these hearts. Others transplant these organs to low risk recipients to reduce the overall risk as the data from United Kingdom Cardiothoracic Transplant Audit steering show that recipient risk is the overriding determinant of immediate post-transplant survival [7]. In our Multi-centre study rejected organs were transplanted to both high and low risk recipients.

The definition of "unacceptable donors" in this study is different from conventional "borderline hearts" in other studies. It does not follow strict criteria and cut-off points for age, smoking, inotropic support and Haemodynamic parameters. Decisions to reject or accept these organs were made on a relative basis with some subjectivity. The presence of multiple centres and organ-sharing scheme provides a positive backup for the best usage of available organs. Some organs will be wasted, if not offered to other centres because of their questionable quality. Our results showed no significant difference in the 30-day mortality, ICU and hospital stay and 5-year survival between the two groups. Although the donors in this study were different from conventional "borderline hearts", our results confirm the findings of the studies, which showed that the outcome with marginal hearts is similar to that obtained with standard donor hearts. In conclusion, our study showed that, hearts declined on medical grounds by one centre can be safely transplanted and should be offered out nationally. The use of these hearts was useful to expand the scarce donor pool and there does not seem to be a justification for denying recipients this extra source of organs. Further work should be done to assess the longer-term results and the quality of life of patients receiving these hearts.
